# Pupil Size Tracks the Effects of Global Context and Semantic Ambiguity on Word-Meaning Processing

**DOI:** 10.5334/joc.454

**Published:** 2025-07-29

**Authors:** Julieta Laurino, Laura Kaczer

**Affiliations:** 1 Laboratorio de Lenguaje y Cognición, Departamento de Fisiología, Biología Molecular y Celular, Facultad de Ciencias Exactas y Naturales, Universidad de Buenos Aires, Argentina; 2 Instituto de Fisiología, Biología Molecular y Neurociencias (IFYBYNE), CONICET, Argentina

**Keywords:** word processing, semantics, discourse processing

## Abstract

Processing word meaning often appears effortless, yet the language system must frequently resolve ambiguity by integrating broad contextual information to ensure comprehension. Understanding the mechanisms underlying the facilitation of global semantic context on word-meaning access remains a key challenge in cognitive neuroscience. In this study, we explore whether global semantic context —specifically, the thematic content of a visually presented short text— reduces the cognitive demands of word-meaning processing. Using pupillometry, we examined the contributions of context congruency and semantic ambiguity across two tasks: a word-association task (Experiment 1) and a semantic relatedness task (Experiment 2). In Experiment 1, global context congruence biased word associations toward context-consistent meanings, and, crucially, this was accompanied by a reduction in pupil size, indicating reduced cognitive effort. Experiment 2 revealed faster and more accurate responses in context-congruent conditions, with a concurrent reduction in pupil size. Notably, the effects of global context on pupil dilation were amplified for more ambiguous words, highlighting an interaction between lexical ambiguity and contextual facilitation. These findings provide new insights into the neurocognitive mechanisms of context-to-word interactions and validate pupillometry as a sensitive marker of cognitive effort during word-meaning processing.

## Introduction

Reading or listening to a word often involves a seemingly effortless access to its meaning. Yet, the vast majority of the words we use are in some way ambiguous (Rodd et al., 2002). Thus, the ability to select a contextually appropriate meaning is a critical component of language comprehension. In such cases, contextual cues —both local and global— play a crucial role in resolving ambiguity and promoting access to the intended meaning. Most research has focused on the function of the local context, which encompasses sentence-level information, grammatical structures, and immediate semantic relationships surrounding the ambiguous word. Studies have consistently demonstrated the facilitatory effects of local context on lexical processing through behavioral measures, such as reduced reading times (Brothers & Kuperberg, 2021; Schustack et al., 1987) and naming latencies (Hess et al., 1995), as well as faster reaction times and increased accuracy in lexical decision tasks (e.g., De Groot, 1985). In addition, electrophysiological markers such as the N400 show a reduced amplitude for contextually facilitated words indicating smoother integration of meaning (Kutas & Hillyard, 1980; Szewczyk & Federmeier, 2022).

Global context, on the other hand, refers to broader thematic or situational frameworks across extended discourse. For instance, the word “bat” might evoke a baseball bat in the context of a sports article or a flying mammal in the context of a wildlife feature. It has been shown that the presence of a global discourse context facilitates processing for globally related concepts (Albrecht & O’Brien, 1993; Carter & Hoffman, 2024; Hess et al., 1995; Schwanenflugel & White, 1991; Vu et al., 2000). In a key study by Hess et al. (1995), participants heard short passages consisting of three to four sentences that served as context, followed by a visually presented target word that they were asked to name. The researchers manipulated the relationship between the target word and two types of context independently: the local context, defined as the final sentence fragment (e.g., “…the English/computer science major wrote the POEM”), and the global context, which referred to the broader situation or scenario described in the preceding sentences (e.g., “The English major met a woman who he was very fond of. He had admired her for a while but wasn’t sure how to express himself. He always got nervous when trying to express himself verbally so …”). Their findings revealed across several experiments that global context facilitated naming times of the target word (e.g., ‘POEM’), whereas local context had no facilitative effect unless it was consistent with the global context.

While it is clear that global context shapes word-meaning access, its precise neurocognitive mechanisms in guiding lexical processing remain less well understood. In terms of neuroimaging, there is a vast body of electrophysiological research focusing on processing of local context in single sentences, whereas fewer studies have used contexts that encompass longer passages beyond a single sentence (Berkum et al., 1999; Boudewyn et al., 2015; Camblin et al., 2007; Federmeier & Kutas, 1999; Helder et al., 2020). Primarily, these studies demonstrate global context effects on the N400, (Kutas & Hillyard, 1980). For instance, in Camblin et al. (2007), participants read sentences (e.g., “Lynn couldn’t stop scratching her arms and LEGS”) where the critical word (‘LEGS’) was either congruent with a preceding passage (e.g., “Lynn had gotten a sunburn at the beach. Nothing she tried would help her dry and irritated skin.”) or incongruent (e.g., “Lynn’s wool sweater was uncomfortable and itchy. She fidgeted as the rough material irritated her skin.”). They found that the congruence of discourse contexts had early and lingering effects on ERP, showing a reduction in N400 amplitudes to target words for congruent conditions.

However, in most of the studies discussed above, it is difficult to completely disentangle local from global influences. This is because the target word is typically presented within a sentence, and although this local sentence is often a controlled or manipulated factor, it remains difficult to fully eliminate the influence of local grammatical and lexical constraints. Additionally, the distinction between what constitutes a local and global context varies across studies, adding further complexity. To address these issues, the present study focuses on analyzing isolated ambiguous words, which lack local contextual information beyond their inherent lexical properties. In addition, we introduced the global context as a separate linguistic component in a different task, preventing it from serving as a local influence. This experimental approach allows us to specifically examine the effects of semantic context on word processing. In particular, we propose an approach that remains relatively underexplored: investigating whether the facilitation effects of global context are linked to a reduction in the neurocognitive demands associated with word processing.

Finally, a number of studies have explored the interaction between contextual constraints and lexical properties (e.g., Rayner et al., 2004; Schwanenflugel & Stowe, 1989). Some of these studies suggest an increased top-down semantic influence, often referred to as semantic control, for less informative word inputs (Hoffman, 2016). Notably, it is proposed that semantic cognition relies on the interaction between the semantic control system and a semantic representation system, which encodes multimodal representations of concepts (Ralph et al., 2017). In this framework, our study looks into the interaction between these two systems, investigating whether global context effects on pupil size are greater for more ambiguous inputs.

### Pupillometry in language research

Pupillometry, which measures the dilation of an individual’s pupil, has long been used as an indicator of brain activity in response to shifts in cognitive load or attentional demands (Beatty, 1982; Kahneman & Beatty, 1966; Sirois & Brisson, 2014). Pupil size was found to be more sensitive and more specific to cognitive effort than behavioral measures (Geller et al., 2016; Kuchinsky et al., 2013; Papesh & Goldinger, 2012; Zekveld et al., 2010). Importantly, the pupillary response is recorded throughout the trial, thereby allowing a more fine-grained, temporal analysis of task-evoked effort that is thought to be a purer index of cognitive effort (Geller et al., 2019). Evidence from electrophysiological and neuroimaging studies suggest that pupil dilation in response to cognitive activity is under the physiological control of the locus coeruleus, the primary source of norepinephrine to the neocortex (e.g., Gilzenrat et al., 2010; Joshi et al., 2016; Murphy et al., 2014). Importantly, norepinephrine has been involved in the modulation of many cognitive functions including memory consolidation and retrieval, working memory, attention, and decision-making (see Sara, 2009, for a review).

Pupillometry has become increasingly used in cognitive language research (Rojas et al., 2024), providing objective and real-time information about the cognitive effort when recognizing, inhibiting, and selecting words (Chapman & Hallowell, 2015; Geller et al., 2016; Guasch et al., 2017; Haro, Guasch, et al., 2017; Haro et al., 2023; Kuchinke et al., 2007; McLaughlin et al., 2022). For example, Kuchinke et al. (2007) recorded pupillary responses while participants performed a lexical decision task (LDT) and found that low-frequency words elicited greater peak pupil dilations and longer reaction times. Interestingly, a few studies have examined the role of context on pupil size during speech comprehension (e.g., Engelhardt et al., 2010; Winn et al., 2015). For instance, in a study of Winn (2016), participants listened to single sentences while the pupillary response was recorded, with varying degrees of context congruence. It was found that semantic context led to rapid reduction of listening effort, suggesting an increased efficiency of ongoing predictive language comprehension.

### Word ambiguity to explore context-to-word interactions

The representation of word meaning in psycholinguistic literature traditionally categorizes words into discrete groups (Cevoli et al., 2021). In this sense, words are deemed unambiguous when there is a one-to-one relationship between the word form and meaning (Lyons, 1981). In contrast, ambiguous words, defined by a one-to-many relationship between form and meaning, are further classified based on the degree of relatedness between their meanings: *polysemous* words have highly related meanings, while *homonymous* words have meanings with little to no relation. For example, the word *bark* can refer to the sound of a dog or the outer layer of a tree, making it a homonym. Importantly, these meanings can either be balanced in frequency or biased, where one meaning is much more common (i.e., dominant) than the other. Also, many accounts propose that word ambiguity lies in a continuum, and that these discrete categories are a useful simplification (Klepousniotou & Baum, 2007). Some even propose that word meanings themselves are extremely context-dependent and continuous, lacking discrete boundaries between them (Trott & Bergen, 2023).

Semantically ambiguous words challenge language comprehension, particularly when listeners must select a less frequent (subordinate) meaning at disambiguation. For biased ambiguous words, dominant meanings are typically accessed more quickly than subordinate meanings (e.g., Rayner & Duffy, 1986; Simpson & Burgess, 1985). Recent studies have shown that preferences for different meanings of ambiguous words are not fixed but rather adaptable based on previous usage information. For instance, Rodd et al. (2013) demonstrated that exposure to sentences that include ambiguous words in a biasing context toward one of their meanings (e.g., “The bark was very loud”) led participants to produce more associated words aligned with that specific meaning. The authors explain these results in terms of an episodic context account where the biasing sentences generate a contextually bound memory representation that aids future comprehension. They further suggest that these episodic representations contribute to the maintenance of situation models (Kintsch, 1988) which facilitate discourse comprehension over longer periods (Curtis et al., 2022; Gaskell et al., 2019).

Challenges in language comprehension arising from semantic ambiguity have also been explored using pupillometry. In particular, Haro et al. (2023) found that ambiguous words led to greater pupillary responses in a number-of-meanings task compared to unambiguous words. In addition, recent results show that pupils dilate more when listening to sentences that include ambiguous words (Kadem et al., 2020).

From these, we suggest that ambiguous words offer a valuable opportunity to explore context-to-word interactions, as the effect of the context can be observed through shifts in meaning access, highlighting how semantic context guides word comprehension.

### This study

In our study, we will address if semantic context reduces the cognitive demands of word-meaning processing. Specifically, we will explore whether and how a global context—i.e., the thematic content of a visually presented short text—can influence the subsequent meaning processing of single words. By using behavioral and pupillometry measurements, our goal is to discern the contributions of word-context congruency and semantic ambiguity on the cognitive load during word-meaning access. To this end, we will employ two complementary tasks with distinct semantic engagement: a word-association task (Experiment 1) and a semantic relatedness task (Experiment 2). Our specific hypotheses are the following: i) pupillary response is modulated by context-to-word matching, such that a smaller pupil size is expected when the context matches the word’s meaning, indicating reduced cognitive load. In addition, this effect will be more pronounced in more engaging semantic tasks; ii) there is a differential effect of contextual modulation on pupil dilation according to the target’s word ambiguity, with more ambiguous words presenting an increased effect of context with respect to non-ambiguous words. This work makes a novel contribution to the literature by examining the specific effect of global semantic context on cognitive demands, as measured by pupillometry, which provides a fine-grained measure of the temporal dynamics of cognitive effort during lexical processing.

## Experiment 1

### Methods

#### Participants

Participants were recruited via mail and laboratory’s social media pages (Twitter, Facebook and Instagram) and signed an informed consent approved by the Ethical Committee of the ‘Instituto de Investigaciones Médicas Alfredo Lanari’. They were all native Spanish speakers with normal or corrected-to-normal vision. Participants received financial compensation for their participation.

Sample size is based on previous literature using the same methodology for the study of word-meaning processing (Guasch et al., 2017; Haro et al., 2023; Kuchinke et al., 2007).

Twenty-five participants took part in the experiment, and two were excluded due to not following the instructions of the experimental task (they wrote the same word presented in the word-association task as response). The final sample consisted of 23 participants (9 females, 13 males and 1 unspecified gender; *M* ± *SD* age in years = 26.1 ± 5.96).

#### Materials

##### Target words

We used 64 Spanish ambiguous words as targets. Ambiguous target words were nouns that belonged to 16 predefined semantic categories (e.g., music, economics, soccer, animals, etc.) (see Appendix A for a full stimuli list and Appendix B for the lexical properties in each experiment). The selection of these target words followed a two-step process. First, we identified words that were either part of the available semantic ambiguity corpora (Fraga et al., 2017; Haro, Ferré, et al., 2017; López Cortés & Horno Chéliz, 2021; Monzó, 1991) or met the criteria of being “potentially ambiguous” defined by Haro et al. (2017): having more than one dictionary entry or more than five dictionary senses in the Spanish Language Dictionary of the Real Academia Española (RAE, 2014).

After identifying a pool of candidate ambiguous words, our second selection criteria was to include ambiguous words that were not strongly biased toward a single dominant meaning, thereby allowing the global context to play a more influential role in meaning selection. To estimate meaning frequency, we followed established methodologies that rely on word-association data (Gilbert & Rodd, 2022; Rice et al., 2019; Twilley et al., 1994) and used on data from the Small World of Words for Rioplatense Spanish (SWOW-RP) database (Cabana et al., 2024). This choice ensured that our estimates were derived from a population of language users closely matching that of our experimental participants. For each ambiguous target word, we manually coded the word-association responses to identify those consistent with the category-relevant meaning. For example, the target word ‘note’ belonged to the music category, and therefore responses such as ‘song’ or ‘listen’ were coded as ‘music-related’ whereas responses such as ‘pen’ or ‘write’ were coded as ‘music-unrelated’. Meaning frequency was thus operationalized as the proportion of responses in SWOW aligned with the semantic category of interest. Across our stimuli, the relative frequency of the category-related meaning averaged 0.35 (SD = 0.17), with values ranging from 0.10 to 0.70. This range ensured that both meanings were sufficiently accessible, allowing for meaningful effects of contextual disambiguation.

Additionally, 32 filler words were selected so that the rationale of the experiment would not be evident to participants. Consequently, these words did not fall into any of the previously defined 16 semantic categories, were not ambiguous and their lexical properties were comparable to those of the target words.

##### Semantic contexts

Sixty-four contexts were used, which consisted of a text paired with an image. Texts and images for these contexts were extracted from Wikipedia articles corresponding to each category (freely available on the OSF). These contexts were representative of the same 16 semantic categories as target words, with four contexts per category. Length ranged from 30 to 35 words (*M* = 33.41, *SD* = 1.60). Crucially, neither the text nor the image contained the target word itself.

Sixteen additional contexts were selected to act as fillers. These were paired with filler words and were representative of another eight categories. Length also ranged from 30 to 35 words (*M* = 33.35, *SD* = 1.66).

#### Procedure

Participants were tested in a medium-illuminated, quiet room. They sat with their head on a chinrest with forehead support, with a distance of 50 cm between their eyes and a 23.8″ computer screen with a resolution of 1920 × 1080 pixels. Participants wore headphones during the whole experiment. Pupil area and eye movements were recorded continuously from both eyes during the task using an eye tracking device, EyeLink 1K (SR Research Ltd.), with a sampling rate of 1000 Hz. PsychoPy software (Peirce et al., 2019) was used to present the stimuli for the experiment. Prior to the experimental trials, a nine-point eye-tracker calibration was conducted.

**Figure 1:**
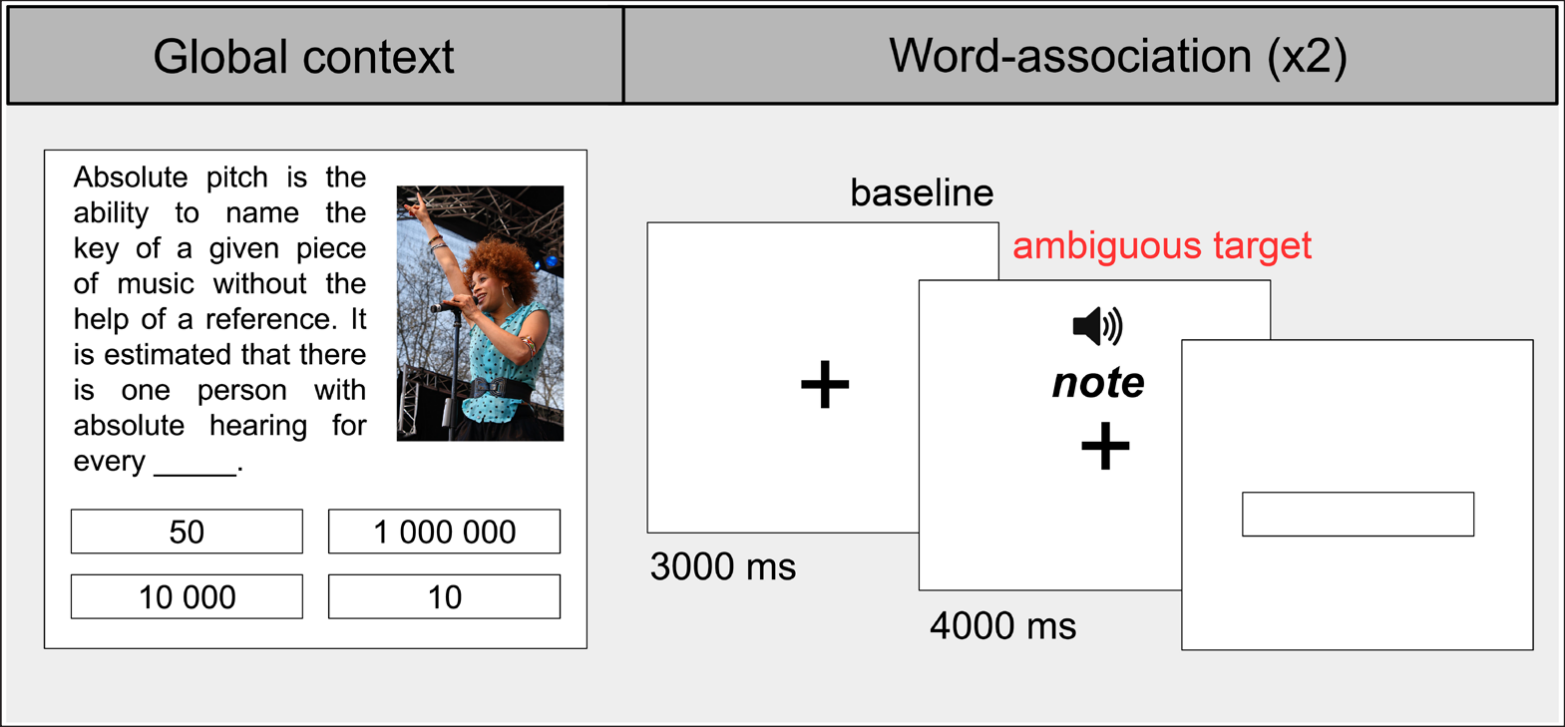
**Experiment 1 example block.** Each global context is followed by two word-associations: one corresponding to the matched condition (e.g., ‘note’) and one to the unmatched condition (e.g., ‘bank’). All words are presented in pseudorandom order across blocks.

Experimental trials consisted of the presentation of the target word in a word-association task that was preceded by a global context. The trials were grouped in blocks, such that two word-association tasks shared the same global context. This way, the duration of the experiment was reduced, controlling for the fatigue that could affect pupil size measures. Each block thus included the presentation of a global context, followed by two word-association tasks (see Figure 1 for an illustrative example).

The global context consisted of the presentation of a short text paired with an image. In order to maintain participants’ attention, the screen had a gamified design, and the text had a missing word that participants needed to complete, earning 1 point for each correct response. Participants had 30 seconds to select the correct option among four, and were given feedback with a tick or a cross appearing on the screen immediately after their response.

In the word-association task, a target or filler word was presented and participants were asked to type in the first word that came to mind upon reading the word. They were instructed to try to avoid giving phrases or sentences as responses. Words were presented in auditory format, contingent with a fixation cross that appeared on screen during baseline and word presentation periods (see Blott et al., 2022, for an auditory word-association task). First, we decided to use auditory and not written stimuli so that the baseline was isoluminant to when the stimuli were shown and, in that way, we controlled for pupil changes derived from luminance changes (Hutton, 2019). Second, small and central stimuli (i.e., a fixation cross) are recommended to avoid the pupil foreshortening effect, which is an apparent change in pupil area that happens when eyes rotate (Hayes & Petrov, 2016). For the auditory stimuli, we used a male Argentinian Spanish neural voice created with Texvoz (www.texvoz.com), a human-like text-to-speech voice generator. The audio of the ambiguous words had a mean duration of 431 ms ± 100 *SD* (Range = 253–675 ms). A fixation cross (black on gray [#808080 Hex] background) appeared on screen 3000 ms before the target or filler word was aurally presented (baseline period) and remained for a total of 4000 ms since the word onset. After the fixation cross disappeared, participants were asked to type their response. The mean duration of the experiment was 32 min.

The experiment began with a single practice block, after which we checked that participants could correctly hear the auditory stimuli. Upon concluding the experiment, participants were required to fill out a final questionnaire. In this questionnaire, they were asked to report their levels of concentration, any inconveniences they may have experienced during the experiment, whether they had noticed any relationship between the two tasks (contexts and word association task), and their attraction/interest towards each of the presented categories. Note that results for the individual category interest are not presented, as they were not the main focus of the present study.

Design: The experiment used a 1 × 2 within-subjects design and included 96 trials, and 48 blocks. In 32 of the blocks, the context was followed by two ambiguous words presented sequentially: one in the matched context condition and the other in the unmatched context condition (pseudorandom order so that in half of the blocks the matched condition appeared first and vice versa). In the matched context condition, the semantic category of the context matched the semantic category of the word (for example, the ambiguous word ‘note’ was preceded by a music-related context). In the unmatched context condition, the category of the context did not match the semantic category of the word. To counterbalance these conditions, the categories were randomly divided into two lists: half of the categories (32 contexts and 32 ambiguous words) appeared in the matched context condition in one list, while the other half appeared in the matched context condition in the other list. Thus, for each participant, an ambiguous word could fall into one of the two context conditions (matched or unmatched). In the 16 remaining blocks, the context was followed by two filler words. The same 32 filler words appeared in both lists and were always preceded by contexts that did not match their semantic category.

##### Online Validation Experiment

An online validation experiment (details and data available in the “Validation experiment” folder on the OSF) was performed to evaluate whether our protocol and stimuli could reliably evidence global context effects on a behavioral task with a large sample of participants (n = 78). The results of this experiment show that the global context—comprising a short text and an image with specific thematic content—preceding an ambiguous word influences meaning preferences in a word-association task. Specifically, the global context biased participants’ associations toward the context-related meaning of the ambiguous word (e.g., following a music-related context, the ambiguous word ‘note’ elicited more associations consistent with its musical meaning). Based on these results, we proceeded to investigate our research questions using pupillometry with the same stimuli and a similar in-lab procedure.

#### Data processing and statistical analysis

Data were analyzed within R (v4.4.0; R Core Team 2024). All models included participants and words as random effects. The structure of random effects was determined by comparing each model’s Akaike’s Information Criterion and with a Likelihood-Ratio Test, using a forward best-path approach (Barr et al., 2013). The significance of the fixed effect was also determined with a Likelihood-Ratio Test comparing the model that includes the factor with a model dropping that factor. The unmatched condition was treated as the reference level and parameters were estimated for the matched condition.

##### Behavior

In the word-association task, responses for target words in each context condition were coded as 1 if they were consistent with the meaning related to the ambiguous word category and 0 if they were not. For example, the target word ‘note’ was classified under the music category, so the response ‘sing’ was coded as 1, while ‘paper’ was coded as 0. Notably, we did not further categorize inconsistent responses to distinguish whether they reflected an alternative meaning or were unrelated, as our main focus was on the modulation of context-matching associations. The proportion of consistent responses in each context condition will be referred to as consistency score.

Consistency data was analyzed with the glmer function from the lme4 package (v1.1.35.3; Bates et al., 2015) to perform a generalized linear mixed model (GLMM) using a binomial family and the logit link, including context as fixed effect (within-participants levels: unmatched and matched). A second model was performed to examine if the effect of the context depended on the relative meaning frequency. This second model included context, relative meaning frequency (between-words quantitative factor), and their interaction as fixed effects. Full specifications of the random effects structures for behavioral models are provided in Appendix C.

##### Pupillometry

For pupil data, the pre-processing guidelines described in Mathôt & Vilotijević (2022) were followed. Data for the left eye is shown here because it better suited a polynomial shape that was used in the Growth Curve Analysis (explained below), but the same pattern of results is observed for both eyes. First, blinks were detected and interpolated using the ‘advanced’ blink-reconstruction mode as implemented in the DataMatrix Python library (v1.0.13; Mathôt, 2024). This algorithm first tries to perform cubic-spline interpolation and, if not possible, performs a linear interpolation. Next, data were downsampled by a factor of 10, from 1000 Hz to 100 Hz. After downsampling, each trial time course was baseline-corrected by subtracting the mean pupil size from the 500 ms before the presentation of the word. Two trial exclusion criteria were applied for the pupil size analysis (note that these trials were not excluded from the word-association analysis). First, a trial was excluded if it contained 40% or more NaN entries from 500 ms prior to word presentation to 4000 ms after word onset (fixation cross disappeared). In total, 19 trials were excluded based on this criterion (1.35% of the trials). Second, we excluded trials with extreme baseline pupil sizes. For each participant separately, baseline pupil sizes were converted to z-scores, and trials where the z-scored baseline pupil size was larger than 2 or smaller than –2 were excluded. This second criterion excluded 63 more trials (4.47% of the total trials). The final number of trials was 1326, with 661 trials in the matched condition and 665 trials in the unmatched condition.

Data analysis for pupil data was performed with the lme4 R package by implementing a Growth curve analysis (GCA). Unlike traditional approaches that use singular value methods such as the mean and peak amplitude, GCA enables a temporal analysis of pupil dilation (Geller et al., 2016; Kuchinsky et al., 2013; McLaughlin et al., 2022; 2024; Winn et al., 2015). It consists of a mixed-effects modeling in which a functional form can be defined to describe non-linear effects of time. In pupillometry, higher-order polynomials are typically a good option to use as a functional form for the curve of a task-evoked pupil response (Mirman, 2014). Therefore, we decided to use GCA because it allows time to be treated as a continuous variable instead of using a time window average, and because it allows the explicit modeling of variations across conditions, individuals, and stimuli (Hershman et al., 2023; McLaughlin et al., 2023).

The time window selected for the GCA analysis was from 500 ms to 3000 ms after the word onset. This time window was selected taking into account the delay of the pupil response and to better suit a polynomial shape while conserving the pupil response curve. Importantly, the time window was decided before conducting the analysis of the effects of interest (Peelle & Van Engen, 2021). The GCA model included fourth-order orthogonal polynomials, context (within-participants levels: unmatched and matched), and their interactions as fixed effects. In this model, the fixed effect of context will determine whether there are differences in overall magnitude between levels (i.e., shifting the curve vertically). On the other hand, interactions between the condition and each polynomial parameter will determine whether the shape of the pupil response differs by condition: the steepness of the slope for the linear component and the sharpness of the peaks for the quadratic, cubic, and quartic components.

### Results

In this first experiment, we asked if global contextual effects have an associated pupillary response, such that a smaller pupil size is expected when the context matches the word’s meaning, indicating reduced cognitive load. For this purpose, the influence of a global context on the subsequent word-meaning preference was evaluated using a word-association task while recording pupillary measures.

**Figure 2:**
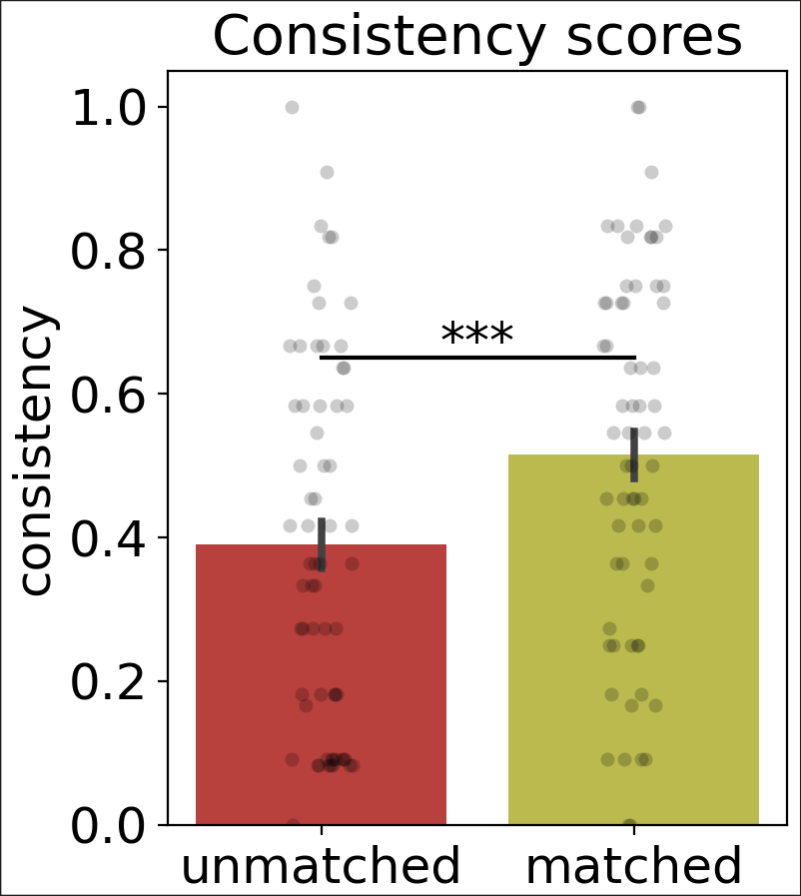
**Experiment 1. Consistency scores of the unmatched and matched context conditions.** Each bar provides the mean (±*SEM*) proportion of associate responses consistent with the context-related meaning across each condition. Points represent the mean consistency for each cue.***p < .0001.

The behavioral results are depicted in Figure 2, showing the mean consistency score for each condition. The unmatched condition presented a consistency score of 0.39. As predicted, this score rose to 0.52 when a thematically congruent context was presented before the target word, that is, in the matched condition. Statistical analysis confirmed that the consistency score difference between the unmatched and the matched context conditions was significant [𝛽 = 0.65, *SE* = 0.12; χ2 (1) = 29.48, *p* < .001] (see Appendix C for a summary of the behavioral statistical models and results.)

In addition, we hypothesized that the effect of semantic context depended on the meaning frequency, such that more modulation would occur for less frequent meanings. However, we found no significant interactions between context and meaning frequency [𝛽 = –0.22, *SE* = 0.76; χ^2^ (1) = 0.08, *p* = .78]. Thus, even for words with a skewed meaning frequency, the context is able to shift the associated responses increasing the consistency with the context-related meaning.

As for the pupillometry analysis, the linear, quadratic, cubic, and quartic polynomials all significantly improved fit (all *p* < .001). As shown in Table 1, including the context condition term on the intercept, linear term, and cubic term improved model fit.

**Table 1:** Experiment 1. Log-likelihood model comparisons for growth curve analysis.


EFFECT	χ^2^	*Df*	*p*

Linear polynomial	9673.6	1	<.001***

Quadratic polynomial	19097	1	<.001***

Cubic polynomial	397.32	1	<.001***

Quartic polynomial	313.74	1	<.001***

Context (levels: unmatched, matched)	65.27	1	<.001***

Context × Linear polynomial	34.99	1	<.001***

Context × Quadratic polynomial	1.10	1	.29

Context × Cubic polynomial	30.44	1	<.001***

Context × Quartic polynomial	.86	1	.35


*Note*. All models included random intercepts for participants and words. Random slopes for the linear, quadratic, and cubic terms were included by participants, and random slopes for the linear, quadratic, cubic, and quartic terms were included by words.

**Figure 3:**
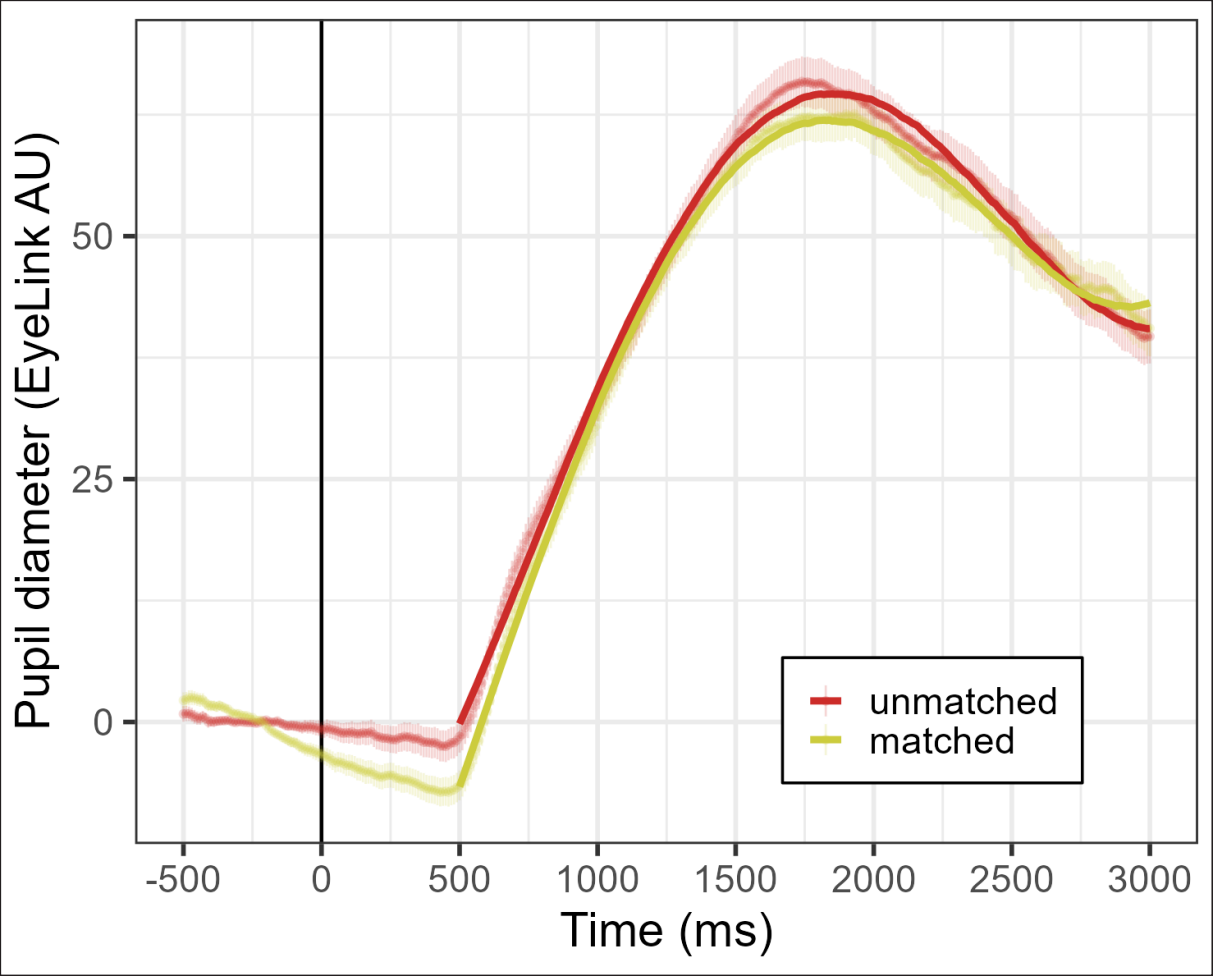
**Experiment 1. Pupil diameter (EyeLink arbitrary units) over time for the unmatched (red) and matched (green) context conditions.** Points represent the raw data means with standard errors, and GCA model fit is overlaid with solid lines. Auditory word onset time is represented as a solid vertical line.

The examination of the dummy-coded main effect of context condition (reference level: unmatched) indicated that the matched condition presented a smaller mean pupil size term (𝛽 = –1.55, *SE* = 0.19, *p* < .001), with a more positive slope term (*β* = 17.92, *SE* = 3.03, *p* < .001), and sharper inflection term (𝛽 = 16.73, *SE* = 3.03, *p* < .001), compared to the unmatched condition (Figure 3).

Thus, it is observed that pupil diameter increases after the presentation of an isolated ambiguous word (auditorily) reaching a peak at around 1800 ms, following a polynomial trajectory. Noteworthy, this increase is modulated by the semantic congruence between previous context and a target word’s meaning. We show that a matched condition results in a smaller overall peak and a different shape of the pupil response curve, compared to an unmatched condition. From these results, it is suggested that global context reduces the neurocognitive demands associated with the processing of an ambiguous word-meaning.

#### Supplemental check measures

Three additional models were performed to check if the context effect depended on: (1) the accuracy of the responses during the global context presentation (i.e., a gamified format was used where participants were required to select a missing word); (2) participants’ awareness of the relationship between the two tasks (as informed during the final questionnaire); (3) the position at which the target word appears (first or second). The first model included context, missing word accuracy (within-participants levels: 0 or 1), and their interaction as fixed factors. The second model included context, awareness (between-participants levels: 0 or 1), and their interaction as fixed factors. The third model included context, target word position (within-words levels: first or second), and their interaction as fixed factors.

For the missing word, 59% of the responses were accurate (above chance level of 25%). The analyses of this factor revealed no significant interactions between context and missing word accuracy [𝛽 = –0.05, *SE* = 0.27; χ^2^ (1) = 0.04, *p* = .84]. Regarding task awareness, 74% of the participants reported they had noticed the relationship between the two tasks. However, we also found no significant interaction between context and awareness [𝛽 = 0.35, *SE* = 0.33; χ^2^ (1) = 1.07, *p* = .30]. Finally, the interaction between context and target word position was also not significant [𝛽 = –0.33, *SE* = 0.24; χ^2^(1) = 1.89, *p* = .17], indicating that the effect of context did not vary depending on the position at which the target word was presented.

These results indicate that responding incorrectly to the missing word, being unaware of the relationship between the tasks or presenting the target word in the second position does not eliminate or diminish the context modulation on meaning selection.

### Discussion

In Experiment 1 we demonstrated global context effects (i.e., a short text and image with specific thematic content) on a word-association task. When the theme of the global context matched one of the meanings of the biased target word (e.g., reading a text related to music and then reading the target word ‘note’), we obtained 13% more responses consistent with the context-related meaning (Figure 2), compared to when they did not match. A similar result was also obtained in the validation experiment, performed online and in a visual modality. These results suggest that word associations can reliably capture meaning shifts according to a previous global semantic context. Importantly, the consistency scores obtained in the unmatched condition are similar to the data from SWOW-RP, a large-scale word-association corpus in Rioplatense Spanish (Cabana et al., 2024), that did not contain any contextual information prior to the cue word. This rules out possible nonspecific effects of the global context on the word-association task, suggesting that the unmatched condition serves as a reliable baseline for our experiments.

Our approach aligns with studies that showed that the interpretation of an isolated ambiguous word in a word association task can be biased by a single encounter with a sentence that includes the ambiguous word in a disambiguating context (Curtis et al., 2022; Rodd et al., 2013). In addition, these studies also observed a biasing effect when using sentences that did not contain the ambiguous word itself but rather a semantically similar word. However, it is worth noting an important methodological difference with our experiments. While the previously mentioned studies use sentences that favor a certain meaning and include the target word itself or a semantically similar word, our experiments use contexts that are broadly thematically related to the target words. We believe that this better reflects the effect of the general thematic of the context, namely the global context, in comparison to the specificity of biasing a particular meaning.

Importantly, we combined the word-association task with a pupillometry measurement. Pupillometric measures, unlike behavioral measures, are time-series data that can track the elevation of effort during a language processing event such as a word presentation and a subsequent verbal response. Our results show that the matched condition presents an overall decreased peak of the pupil response curve, compared to an unmatched condition (Figure 3). From this, it is suggested that global context mitigate the cognitive effort required to process ambiguous words. We consider that this result is particularly striking, given the open-ended nature of the task and despite the absence of explicit instructions of semantic engagement.

Regarding the dynamics of the pupil response (Figure 3), we observe an early divergence between conditions starting around 500 ms and a later re-emergence of the effect around 1500 ms. This pattern likely reflects distinct components of word processing, such as early word recognition and later semantic activation. Therefore, the early divergence may indicate facilitated recognition when the global context supports the intended meaning, while the later effect may correspond to more efficient meaning access or semantic integration for context-matching interpretations. We will expand on this interpretation in the General discussion.

To address whether there is a differential effect of context according to the target’s word ambiguity, the next experiment will also include a set of non-ambiguous words. Moreover, we will use a semantic relatedness task to assess word processing. The rationale for this decision is that the word-association task imposes some practical limitations, as its relatively slow nature hampers the correlation between pupillometry and behavioral measurements. In addition, this task does not force participants to choose a specific word-meaning and it is unsuitable for addressing changes in meaning access when words are not ambiguous. We suggest that a task that involves a deeper semantic processing would elicit larger semantic context effects in the pupillary response (see Haro et al., 2023 for a comparison of different tasks regarding semantic engagement). Thus, in the following experiment, we use a semantic relatedness task that encourages participants to give a fast and correct response, increasing the sensibility of the experimental measurement and reducing response bias.

## Experiment 2

### Methods

#### Participants

Participants were recruited via mail and laboratory’s social media pages (Twitter, Facebook and Instagram) and signed an informed consent approved by the Ethical Committee of the ‘Instituto de Investigaciones Médicas Alfredo Lanari’. They were all native Spanish speakers with normal or corrected-to-normal vision. Participants received financial compensation for their participation.

Thirty-four participants took part in the experiment, and two were excluded due to low performance in the semantic relatedness task (below 70% accuracy). The final sample consisted of 32 participants (15 females, 3 males and 14 unspecified gender; *M* ± *SD* age in years = 21.9 ± 2.94).

#### Materials

##### Target words

In Experiment 2, we included both ambiguous and non-ambiguous target words. To avoid increasing the experiment’s length, we selected a subset of 32 words from the 64 ambiguous target words used in Experiment 1. For each of the 16 categories in Experiment 1, we chose the two of the four ambiguous words that exhibited the largest differences in consistency between conditions. The 32 non-ambiguous target words belonged to the same 16 semantic categories as ambiguous words. To create this set, we selected words that were either labeled as non-ambiguous in the available semantic ambiguity corpora or that met the criteria of being “potentially unambiguous” as defined by Haro et al., (2017) (i.e. words that had one dictionary entry and five or fewer dictionary senses in the Spanish Language Dictionary of the Real Academia Española). Additionally, as in the previous experiment, 32 filler words were used. Finally, to act as probes in the semantic relatedness task we selected a set of items related to the target words (i.e., related probes), and also a set unrelated to the filler words (i.e., unrelated probes). We selected nouns with similar lexical properties to the target words, not occurring in the global context, not a synonym of the target word (see Appendix B).

##### Global semantic contexts

The same global contexts as in Experiment 1 were used.

#### Procedure

Participants were tested in the same room with the same set up as in Experiment 1.

**Figure 4:**
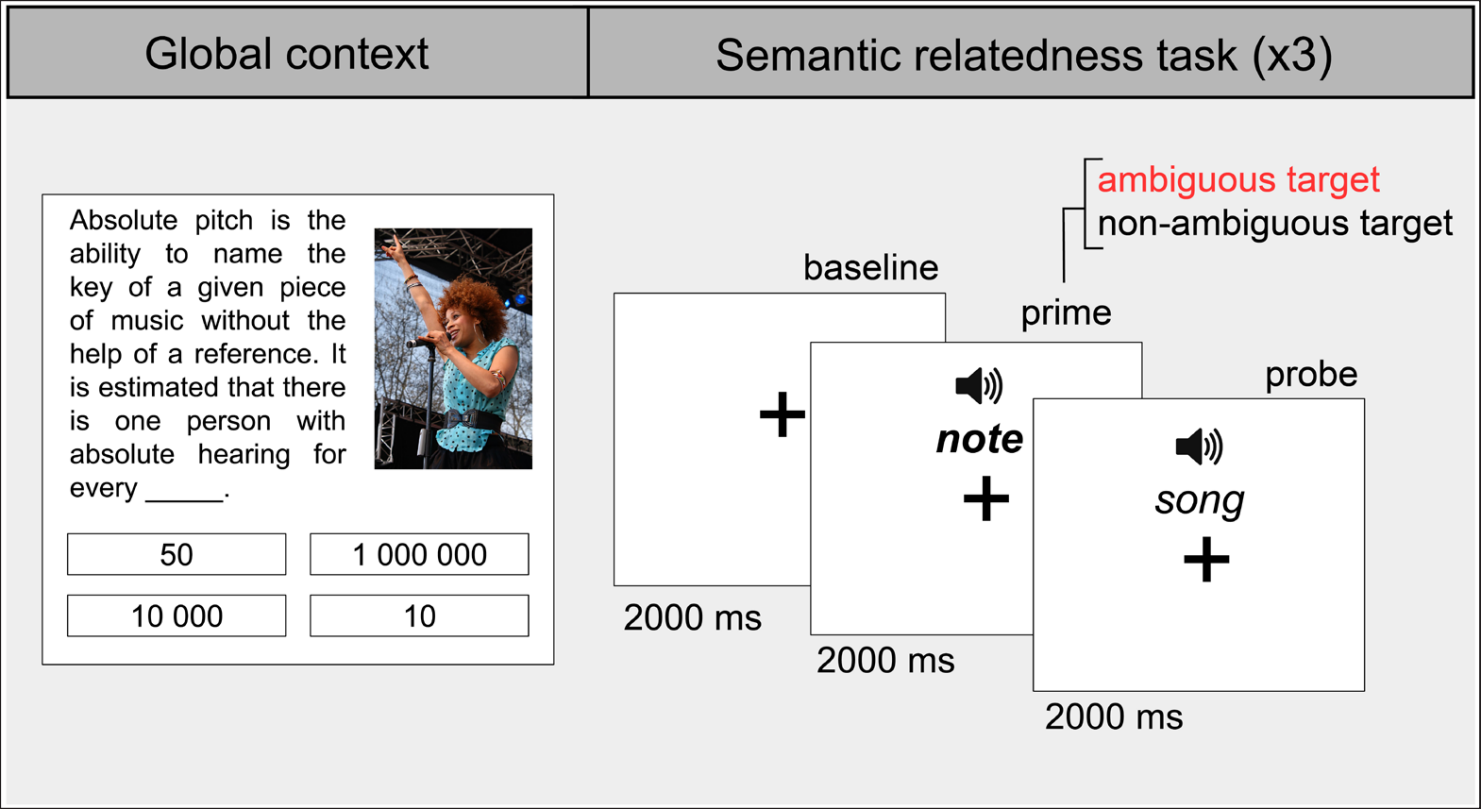
**Experiment 2 example block.** Each global context is followed by three semantic relatedness pairs: one corresponding to the matched context condition, one to the unmatched context condition, and one filler. All words are presented in pseudorandom order across blocks.

Experimental trials consisted of the presentation of the target word in a semantic relatedness task that was preceded by a global context. To minimize experiment duration and participant fatigue, trials were grouped into blocks, with three relatedness tasks sharing the same global context. Each block thus included the presentation of a global context, followed by three sequential semantic relatedness tasks (see Figure 4 for an illustrative example).

The presentation of the global context followed the same procedure as Experiment 1.

In the semantic relatedness task, participants were instructed to decide as quickly and accurately as possible whether a pair of sequential words (i.e., prime–probe) were related or not. Ambiguous (e.g., ‘note’), non-ambiguous (e.g., ‘trumpet’), and filler (e.g., ‘mirror’) words were used as primes. For ambiguous and non-ambiguous targets, related words (e.g., ‘note-SONG’ and ‘trumpet-WIND’) were used as probes, while unrelated words were used as probes for fillers (e.g., ‘mirror-DIRECTOR’). For the auditory stimuli, we used a male Argentinian Spanish neural voice created with Texvoz (www.texvoz.com), a human-like text-to-speech voice generator. The audio of the ambiguous words had a mean duration of 430 ms ± 100 *SD* (Range = 253–606 ms) and the audio of the non-ambiguous words had a mean duration of 494 ms ± 88 *SD* (Range = 365–805 ms). A fixation cross (black on gray [#808080 Hex] background) appeared on screen 2000 ms before the ambiguous, non-ambiguous, or filler prime was aurally presented (baseline period), and remained until the end of the semantic relatedness task. Two seconds after the prime onset, participants heard the probe and had to make a ‘yes’ or ‘no’ response. If participants did not respond after 2000 ms from the probe onset passed, the fixation cross turned red, indicating that response was slow. The responses were made with a button press from the keyboard, using the ‘j’ key for the ‘yes’ responses and the ‘k’ key for the ‘no’ responses. The mean duration of the experiment was 18 min.

The experiment began with two practice blocks, after which we checked that participants could correctly hear the auditory stimuli. Upon concluding the experiment, participants were required to fill out the same final questionnaire as in Experiment 1.

Design: The experiment used a 2 × 2 within-subjects design and comprised 96 trials, and 32 blocks. On half of the blocks, the context was followed by two ambiguous words with its related probes—one in the matched context condition and the other in the unmatched context condition— and by one filler word with its unrelated probe. On the other half of the blocks, the context was followed by two non-ambiguous words with their related probes —one in the matched context condition and the other in the unmatched context condition— and by one filler word with its unrelated probe. The order of the three semantic relatedness tasks was pseudorandom so that in one-third of the blocks the matched condition, unmatched condition, and fillers appeared first, second, and third. To counterbalance these conditions, the categories were randomly divided into two lists: half of the categories (32 contexts, 16 ambiguous words, and 16 non-ambiguous words) appeared in the matched context condition in one list, while the other half appeared in the matched context condition in the other list. The same 32 filler words appeared in both lists. Thus, for each participant, a target word could fall into one of the two context conditions (matched or unmatched). Note that while filler words were always paired with unrelated probes, ambiguous and non-ambiguous target words were always paired with related probes.

#### Data processing and analysis

Data were analyzed within R (v4.4.0; R Core Team 2024). All models included participants and words as random effects. The structure of random effects was determined by comparing each model’s Akaike’s Information Criterion and with a Likelihood-Ratio Test, using a forward best-path approach (Barr et al., 2013). The significance of the fixed effect was also determined with a Likelihood-Ratio Test comparing the model that includes the factor with a model dropping that factor. Pairwise comparisons were performed using the emmeans package in R (v1.10.1; Lenth, 2024). The unmatched condition and the non-ambiguous condition were treated as the reference level and parameters were estimated for the matched and ambiguous conditions.

##### Behavior

In the semantic relatedness task, accuracy data were analyzed with the glmer function from the lme4 package to perform a generalized linear mixed model (GLMM) using a binomial family and the logit link. Response times (RTs) were analyzed with the lmer function from the lmerTest package (v3.1.3; Kuznetsova et al., 2017) to perform a linear mixed model (LMM). They were measured from probe onset. Only accurate trials were considered for this analysis (discarding 11.4% of the data). Also, trials with response times less than 300 ms were discarded (0% of the data) while the upper limit was the end of the trial (2000 ms). The response times were log10-transformed [logRT = log10(RT)] as visual inspection of a residuals versus fitted values plot and a histogram of residuals revealed deviations from normality. After transforming the RTs, any logRTs that were more than two standard deviations above or below each participant’s mean per condition were removed (3% of the remaining data). The models for accuracy data and logRTs included context (within-participants levels: unmatched and matched), word type (within-words levels: non-ambiguous and ambiguous), and their interaction as fixed effects. Finally, for both accuracy and logRTs data, we performed a model that included context and relative meaning frequency (between-words quantitative factor), and their interaction as fixed effects. Full specifications of the random effects structures for behavioral models are provided in Appendix C.

##### Pupillometry

For pupil data, the same pre-processing guidelines and trial exclusion criteria as Experiment 1 were followed. First, 39 trials were excluded because of containing 40% or more NaN entries from 500 ms prior to word presentation to 2000 ms after word onset (start of probe) (2.18% of the trials). Second, 69 more trials with extreme baseline pupil sizes were excluded (3.85% of the total trials). Note that these trials were not excluded from the semantic relatedness task analysis. Finally, only trials that were accurate in the semantic relatedness task were considered for the pupillometry analysis (discarding 9.03% of the remaining data). The final number of trials was 1532, with 389 trials in the matched and 346 in the unmatched condition for ambiguous words, and 402 trials in the matched and 395 in the unmatched condition for non-ambiguous words.

The time window selected for this analysis was from 500 ms to 2000 ms after the stimulus onset. The GCA model included third-order orthogonal polynomials, context (within-participants levels: unmatched and matched), word type (within-words levels: non-ambiguous and ambiguous), and their interaction as fixed effects.

Finally, we evaluated whether pupil size was predictive of behavioral measurements. We performed a linear mixed model (LMM) for logRTs, as explained previously, which in this case only included mean pupil size (averaged across time for each trial) as fixed effect. The pupil size time window selected for this analysis was the same as in the GCA (500 ms to 2000 ms after the stimulus onset).

### Results

We asked if global context reduces a word’s processing time, using a semantic relatedness task with pupillary measurements. Moreover, we evaluated if more ambiguous words present greater context effects with respect to non-ambiguous words. Thus, the factorial design includes word type (ambiguous and non-ambiguous words), as well as context-to-word congruence (unmatched and matched) comparisons.

**Figure 5:**
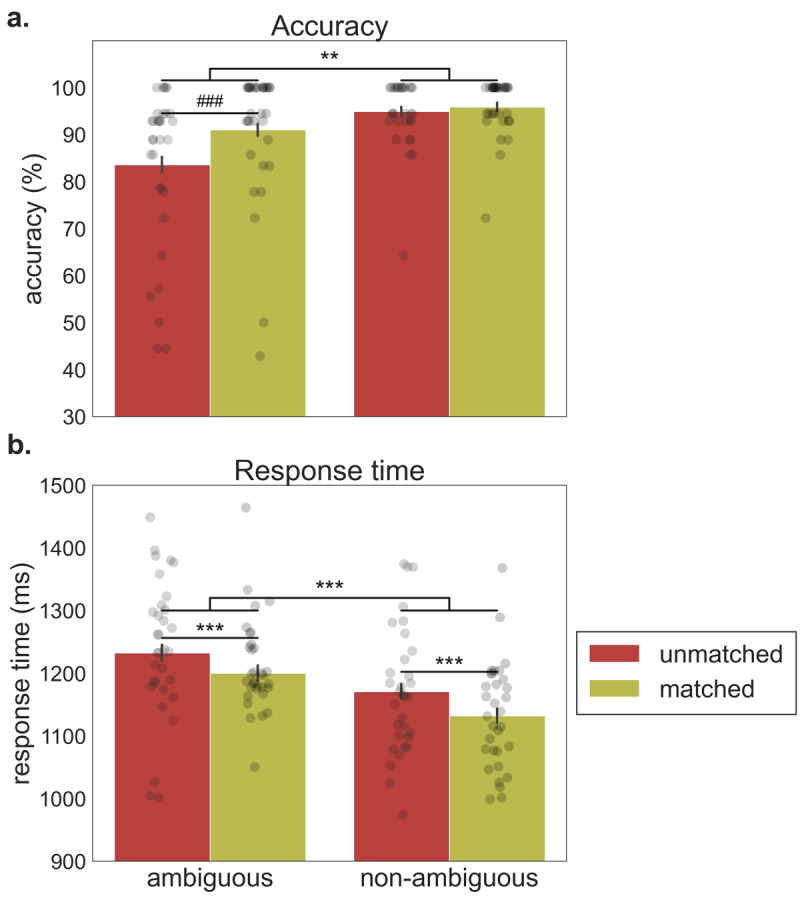
**Experiment 2. Performance on the semantic relatedness task for ambiguous and non-ambiguous words of the unmatched and matched context conditions.** Each bar provides the mean (±*SEM*) across each condition. Points represent the mean for each cue. **a)** Accuracy (percentage of correct prime-probe relatedness judgments). ^###^*p* < .001 (pairwise simple contrasts); **p < .01 (main effect). **b)** Response times (in milliseconds) for correct responses.***p < .001 (main effect).

The analysis of accuracy data (see Figure 5a) presented a non-significant interaction between context and word type [𝛽 = 0.53, *SE* = 0.38; χ^2^(1) = 1.90, *p* = .17], and a main effect of context [𝛽 = 0.65, *SE* = 0.18; χ^2^(1) = 13.68, *p* < .001] and word type [𝛽 = –1.11, *SE* = 0.34; χ^2^(1) = 10.20, *p* = .0014], such that the matched condition was more accurate than the unmatched condition and the ambiguous words were less accurate than the non-ambiguous words (see also Appendix C for a summary of the behavioral statistical models and results). However, since we were particularly interested in evaluating if the effect of context differed between ambiguous and non-ambiguous words, we tested the simple effect of context for both types of words. Pairwise simple contrasts indicated that ambiguous words presented more accurate responses for the matched condition than for the unmatched condition (𝛽 = 0.82, *SE* = 0.22, *p* < .001), while non-ambiguous words presented no significant differences in accuracy between the unmatched and matched context conditions (𝛽 = 0.30, *SE* = 0.31, *p* = .36).

The analysis of response times (see Figure 5b) presented a non-significant interaction between context and word type [𝛽 = 0.00, *SE* = 0.02; *F*(1, 59.59) = .02, *p* = .89], and a main effect of context [𝛽 = –0.03, *SE* = 0.01; *F*(1, 60.56) = 12.99, *p* < .001] and word type [𝛽 = 0.06, *SE* = 0.02; *F*(1, 58.02) = 15.81, *p* < .001], indicating that the matched condition was faster than the unmatched condition and that the ambiguous words elicited slower responses than the non-ambiguous words. In this case, pairwise simple contrasts indicated that the matched condition presented faster responses for both the ambiguous (*β* = –0.03, *SE* = 0.01, *p* = .010) and non-ambiguous words (𝛽 = –0.04, *SE* = 0.01, *p* = .020).

When focusing on the effects of the relative meaning frequency of ambiguous words on accuracy data, we found no significant interaction with context [𝛽 = –1.96, *SE* = 1.72; χ^2^(1) = 1.29, *p* = .26]. As expected, there was a main effect of context [𝛽 = 0.81, *SE* = 0.22; χ^2^(1) = 13.19, *p* < .001], as well as a significant main effect of relative meaning frequency [𝛽 = 4.90, *SE* = 1.57; χ^2^(1) = 9.68, *p* = .002], with more frequent meanings being more accurate. In the case of response times, the interaction between context and relative meaning frequency was significant [𝛽 = 0.27, *SE* = 0.09; *F*(1,29.10). = 9.72, *p* = .004], such that the effect of context was greater for less frequent meanings. Therefore, contrary to previous results with word associations, here we found that the relative meaning frequency had an impact on the context modulation of the semantic relatedness task response times.

As for the pupillometry analysis, the linear, quadratic, and cubic polynomials all significantly improved fit (all *p* < .001). As shown in Table 2, including the context on the intercept and linear term improved model fit.

**Table 2:** Experiment 2. Log-likelihood model comparisons for growth curve analysis.


EFFECT	χ2	*Df*	*P*

Linear polynomial	1557.1	1	<.001***

Quadratic polynomial	1958.8	1	<.001***

Cubic polynomial	156.34	1	<.001***

Context (levels: unmatched, matched)	473.72	1	<.001***

Word type (levels: non-ambiguous, ambiguous)	1.18	1	0.28

Context × Word type	159.17	1	<.001***

Context × Linear polynomial	15.43	1	<.001***

Context × Quadratic polynomial	2.46	1	.12

Context × Cubic polynomial	1.17	1	.28

Word type × Linear polynomial	.37	1	.54

Word type × Quadratic polynomial	3.61	1	.058

Word type × Cubic polynomial	.03	1	.86


*Note*. All models included random intercepts for participants and words, and random slopes for the linear, quadratic and cubic terms by both participants and words.

**Figure 6:**
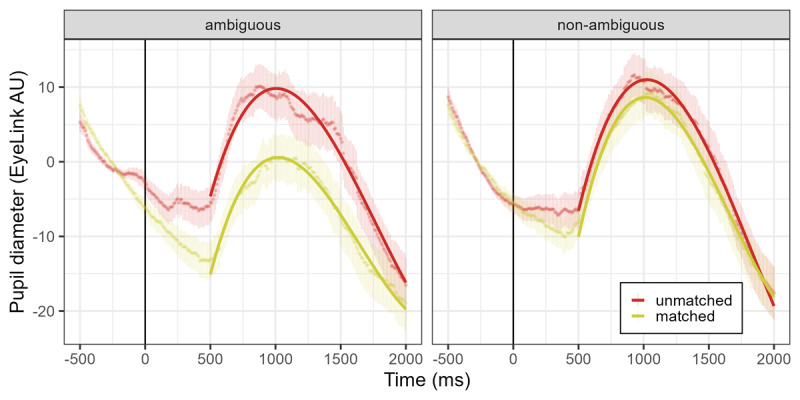
**Experiment 2. Pupil diameter (EyeLink arbitrary units) over time for ambiguous and non-ambiguous words of the unmatched and matched context conditions.** Points represent the raw data means with standard errors, and GCA model fit is overlaid with solid lines. Auditory word onset time is represented as a solid vertical line.

The examination of the dummy-coded main effect of context (reference level: unmatched) indicated that the matched condition presented a smaller mean pupil size (𝛽 = –2.44, *SE* = 0.35, *p* < .001) and a more positive slope term (𝛽 = 12.08, *SE* = 3.07, *p* < .001), compared to the unmatched condition. Importantly, Table 2 shows that the interaction between context and word type also significantly improved model fit (*p* < .001), indicating that the effect of context was larger for ambiguous words (Figure 6, left panel), compared to non-ambiguous words (𝛽 = –6.33, *SE* = 0.50, *p* < .001) (Figure 6, right panel).

Finally, we were interested in evaluating if the reduction in pupil dilation was associated with a faster response time. The analysis revealed non-significant effects of mean pupil size on response times [𝛽 = 0.00, *SE* = 0.00; *F*(1, 1379.3) = .61, *p* = .44]. Therefore, our data suggests that pupil size does not account for the behavioral effects obtained in the semantic relatedness task.

#### Supplemental check measures

We further checked that the context effect did not depend on participants’ awareness of the relationship between the two tasks (as informed during the final questionnaire) or on the position at which the target word appears (first, second or third). For both accuracy and logRT data, the first model included context, awareness (between-participants levels: 0 or 1), and their interaction as fixed effects. The second model included context, target word position (within words levels: first, second or third), and their interaction as fixed effects.

Task awareness for this task was lower than word-association (Experiment 1), with only 29% of the participants reporting they had noticed the relationship between the two tasks. We found no significant interaction between context and awareness for accuracy [χ^2^ (1) = 0.35, *p* = .55] and response times [*F*(1,1765.2) = 0.91, *p* = .34]. Similarly, there were no significant interactions between context and target word position for accuracy [χ^2^(1) = 3.30, p = .19] or response times [F(2, 67.38) = 2.27, p = .11]. These results indicate that, as in Experiment 1, neither participants’ awareness of the task relationship nor the position of the target word influenced the contextual effects.

### Discussion

Overall, results of Experiment 2 demonstrate that word meaning processing is influenced by both context congruence and ambiguity, as well as by their interaction. Specifically, a matching context reduces response times and increases precision, while ambiguity has the opposite effect. On a finer grain, accuracy data show a greater effect of context for ambiguous words. Regarding pupillometry, and consistent with the findings from Experiment 1, matched contexts led to reduced pupil dilation during word processing. Moreover, supporting our hypothesis that tasks requiring deeper semantic processing—such as the semantic relatedness task—would elicit stronger semantic context effects in pupillometry, we observed larger effect sizes in this experiment compared to Experiment 1, which uses a word-association task. Importantly, pupil dilation indicated an interaction between context congruence and ambiguity, highlighting that contextual effects were more pronounced for ambiguous words compared to non-ambiguous ones.

We were able to observe a global context effect on behavioral and pupillometry measures for both ambiguous and non-ambiguous words. Nonetheless, accuracy and pupil size differences due to global context were significantly greater for ambiguous compared to non-ambiguous words. A useful parallel can be drawn between these results and studies that look into the interaction between the representational features of the word (in our case, word ambiguity) and control mechanisms (i.e., the thematic context). Previous work suggested that less informative inputs are more sensitive to executive processes that provide top-down regulation of knowledge (Hoffman, 2016), often referred to as semantic control. From this perspective, higher levels of ambiguity would imply more meaning variability and, therefore, the global context will be of special importance to overcome the associated uncertainty (see Schwanenflugel et al., 1988, for a similar proposal). Additionally, the observed interaction between context and ambiguity provides support for an interactive involvement of both control and representation systems during word processing, rather than these factors being involved in an additive and sequential manner.

The comparison between ambiguous and non-ambiguous words presented a mixed pattern of results. Behavioral results in Experiment 2 showed a disadvantage for ambiguous words in terms of accuracy and response times. This is in line with several studies showing a disadvantage for homonyms compared to non-ambiguous words both in lexical and semantic decision tasks (Armstrong & Plaut, 2008; Brown, 2008; Rodd et al., 2002). Pupillometry results, on the other hand, indicate that ambiguous words themselves do not require additional cognitive effort, in contrast with the studies of Haro et al. (2023) and Kadem et al. (2020). That is, results from Experiment 2 (Figure 6) show that ambiguity has no significant main effect on pupil dilation.

Regarding our behavioral results, it is interesting to discuss the possible cognitive mechanism that leads to a disadvantage in homonym processing. Some studies argue that comprehension delays occur because the separate semantic representations of ambiguous words, particularly homonyms, compete for activation in a neutral context or in isolation (Beretta et al., 2005; Rodd et al., 2002). An alternative account proposes that the ambiguity disadvantage stems from decision-making difficulties during semantic categorization tasks (Hino et al., 2006; Pexman et al., 2004). The different meanings of ambiguous words often belong to different categories, creating response conflicts. Under the decision-making account, delays arise due to the demands of task-specific response selection, with semantic representations activated independently, and without interference. We suggest that our behavioral results align more closely with the idea that the ambiguity disadvantage arises from conflicts in decision-making rather than semantic competition. Along this line, we only see the ambiguity disadvantage when a semantic decision has to be made after listening to the probe.

The measure of pupil size during word processing, instead, does not show an ambiguity effect, probably because participants did not listen to the probe yet and thus cannot make a decision. Our results differ from studies from Haro et al. (2023) and Kadem et al. (2020), which found an effect of semantic ambiguity on pupil size. Several methodological aspects could account for this discrepancy in the effects observed. First, the tasks used in both studies were very different from the ones employed in our experiment. For instance, Haro et al. (2023) found an ambiguity effect on pupil size in a number-of-meanings task, where participants had to indicate if a written word had one or more meanings. This is a task that, besides being the stimuli presented in written format (vs. aurally in our experiment), involves very high levels of semantic processing and induces explicit awareness of the multiplicity of meanings of ambiguous words. In fact, they found no pupillary response differences between ambiguous and non-ambiguous words when using a semantic categorization task and a lexical decision task, tasks that do not require the simultaneous activation of all meanings and/or are less semantically engaging. In the case of Kadem et al. (2020), words were not presented in isolation but rather embedded in auditory sentences that contained or not ambiguous words. Therefore, changes in pupil size reflect cognitive demands related to sentence comprehension rather than single-word processing.

A further methodological consideration is the selection of ambiguous words. Unlike Haro et al. (2023) which used number-of-meanings ratings, and Kadem et al. (2020) which used dictionary entries (and a different language, i.e. English), we used a scoring derived from a word association task to select ambiguous words. Although all of these measures are positively correlated (Haro, Ferré, et al., 2017), from our perspective, word associations present several advantages over dictionaries and other subjective measures of word ambiguity. First, dictionaries include meanings that are unknown or unused by the majority of the speakers and are not frequently updated to include novel meanings (Haro & Ferré, 2018). Subjective measures are an alternative to this limitation and they also allow for the estimation of relative meaning frequency. We find, however, that the use of number-of-meanings ratings as a subjective measure also poses limitations because, as mentioned before, it requires explicit awareness of the multiplicity of meanings of ambiguous words, a requirement that is far from their natural usage and that could lead to an overestimation of the frequency of certain meanings. Word associations, in contrast provide us with a more unbiased measure of meaning, that gives us access to common sense and experiential features of meaning (De Deyne et al., 2021; Liu et al., 2022; Nelson et al., 2004). Lastly, in addition to the advantages mentioned above, using the SWOW-RP word associations database to determine ambiguity scores allowed us to account for the local usage of the words in our Rioplatense Spanish population, as such local norms were not available in other existing corpora.

Finally, the correlation between pupillometry and behavior indicated that pupil size measures were not predictive of response times. This may suggest that the two measures capture distinct aspects of word processing, potentially reflecting different processing stages (see Kadem et al., 2020, for similar results using a semantic relatedness task). Alternatively, as suggested by an anonymous reviewer, pupil dilation may represent an integrated signal of partially overlapping cognitive processes, not necessarily tied to discrete processing stages. In any case, we propose that pupil size serves as a valuable index of neurocognitive activity during context-to-meaning integration, offering complementary insights alongside behavioral measures.

## General discussion

In the present study, we consistently demonstrated that global semantic context facilitates the processing of related meanings and modulates pupillary responses accordingly, suggesting a reduction in cognitive load. This was evidenced, first, in a word association task (Experiment 1) where participants biased the responses towards a context-related meaning. Notably, during word processing, the pupil size was reduced when the context and a target word matched semantically. These effects were also observed in a semantic relatedness task, which required participants to choose a specific word-meaning (Experiment 2). More accurate and faster responses, along with a reduction in pupil size, evidenced the processing facilitation generated by the global semantic context. Furthermore, this experiment also showed that these effects are not specific to ambiguous words, as words with lower ambiguity also presented faster response times and reduced pupil size in the presence of a congruent global context. However, data suggests that stronger effects of context might be present as the ambiguity of the words increases.

### The role of global context

Congruent global context biased responses toward related meanings and resulted in more accurate and faster response times, consistent with evidence showing global context-based facilitation in behavioral (Hess et al., 1995; Vu et al., 2000), reading time (Albrecht & O’Brien, 1993; Camblin et al., 2007; Carter & Hoffman, 2024; Schwanenflugel & White, 1991) and neurophysiological studies. Notably, most previous studies showing a facilitation effect of the global context used target words in individual sentences preceded by context sentences (Berkum et al., 1999; Camblin et al., 2007; Carter & Hoffman, 2024). Alternatively, in our study, the facilitation effect was observed for target words that were presented in isolation, allowing us to evidence the effect of global semantic context free from any constraints and influence of the local sentence. Moreover, the context and the target words were presented in two very different tasks, and no explicit link between the two was given to the participants during instructions. This design choice was intended to reflect a more implicit and strategy-free effect of global context. Even so, it is possible that participants might have become aware of the link between the tasks and adopted a specific strategy for selecting the related meaning. However, we think it is unlikely that this explains the global context effects because our measure of explicit awareness obtained in the final questionnaire showed no effects on participants’ responses (see Supplemental check measures in the Results section). Therefore, we suggest that even if participants were not consciously aware of the thematic relation between the context and the target word, the effects of the global context were still evident in their responses (see Graves et al., 2021, for a similar proposal where uncertainty is detected in pupil size but remains inaccessible to conscious awareness).

Experimental protocols used in our study pose yet another difference with previous studies evaluating global context effects: the sensory modality employed. We used a mixed modality approach, where the context was presented in a written format, and the target word was presented in an auditory format. Remarkably, the global context effect on meaning selection observed in the validation experiment, when both tasks shared modalities, was almost identical to that in Experiment 1, where the modality changed. This result is important because it extends the literature demonstrating cross-modal influences in word processing (e.g., Gilbert et al., 2018; Holcomb & Anderson, 1993; Swinney, 1979).

Additionally, we investigated whether the global context effect in the ambiguous words was modulated by the relative meaning frequency. That is, it is possible to conceive that contextual facilitation occurs only in cases of low-frequency meanings. This could reflect that contextual modulation is highly informative when the meaning is less common. In Experiment 1, word-association responses did not show a modulation according to relative meaning frequency. However, in Experiment 2, which used a semantic relatedness task, we found that global context effects were indeed modulated by relative meaning frequency. Response times indicated that facilitation was greater when the biased meaning was less frequent. The difference between experiments may be attributed to the sensitivity of the behavioral tasks; word-association tasks are self-paced, whereas semantic judgment tasks require quick responses. Regarding the implication of our results, we suggest they can be informative for studies that take lexical ambiguity as an explanatory variable, as we showed a gradual effect of relative meaning frequency. Thus, it might be useful to treat word ambiguity as a continuous variable rather than a binary property (see the proposal of Rodd, 2020).

Finally, it is interesting to discuss our results in the framework of several theories of language comprehension that seek to explain context effects on word processing. On the one hand, predictive accounts emphasize the brain’s ability to anticipate the likely meaning of an upcoming word based on its linguistic environment (e.g., Fleur et al., 2020; Kutas & Federmeier, 2011). These predictive processes are thought to enhance efficiency and accuracy in lexical access, ultimately promoting smoother comprehension. In this line, the ‘proportional preactivation account’ (Brothers & Kuperberg, 2021) suggests that words are pre-activated in proportion to their estimated probability of occurrence. Contextual facilitation, in turn, arises when pre-activated semantic representations align with the representations of incoming target words (DeLong et al., 2005; Kleinman et al., 2015; Szewczyk & Federmeier, 2022). Our findings, particularly the reduction in pupil dilation for context-congruent and ambiguous words, can be aligned with this view by suggesting that predictive mechanisms reduce cognitive effort during word processing. In contrast, integration-based accounts propose that comprehension relies on forming and updating a coherent event-based mental representation, drawing on information from broader discourse (Kintsch, 1988; Zwaan & Radvansky, 1998). This process involves leveraging schemata activated by semantic cues to access related information stored in long-term memory (Sharkey & Mitchell, 1985). The observed interplay between global context and lexical ambiguity in our study also aligns with these accounts, indicating that global thematic frameworks guide the integration of ambiguous words into broader discourse structures. Together, our findings suggest that predictive and integrative processes may operate in tandem, with global context facilitating both pre-activation and integration to optimize word-meaning access.

### Pupillometry as a window into real-time language processing

In line with several previous pupillometry studies (Geller et al., 2016; Guasch et al., 2017; Haro et al., 2023; Kuchinke et al., 2007), we found that pupil size is sensitive to word-meaning access. Specifically, our findings indicate that a smaller pupil diameter is observed in the presence of congruent contextual information.

It is broadly established that changes in pupillary response indicate increased processing load, and this possibly includes a combination of several cognitive functions such as attention, engagement, arousal, and effort (Winn et al., 2018). As previously discussed, context facilitation is suggested to result from the overlap between pre-activated and incoming semantic representations (e.g., Brothers & Kuperberg, 2021). Along this line, we propose that this overlap leads to the recruitment of fewer cognitive resources, thereby reducing the effort required during word processing.

Although pupillometry lacks a definitive characterization and isolation of the distinct stages underlying linguistic processing, recent work suggests that it is likely that there are different dilation components related to word recognition, semantic activation and lexical retrieval (Rojas et al., 2024). Building on this, we offer a preliminary interpretation of the pupil response dynamics. In both experiments, an early divergence between conditions emerged around 500 ms. This divergence may reflect facilitated word recognition when the global context supports the intended meaning. In line with this, Pluchino et al. (2014) reported earlier pupil responses (~280 ms) in semantically related conditions, and suggest that semantic congruence speeds up early perceptual processing—a stage they distinguish from later processes involved in detecting and evaluating semantic associations. On the other hand, the later re-emergence of the pupil size difference in Experiment 1—and its sustained presence in Experiment 2—may reflect word-meaning access or semantic integration, which are more efficient for context-matching meanings. However, we cannot rule out the possibility that other cognitive processes may also contribute to the observed pupillary effects.

In fact, another useful framework to interpret the observed dilation dynamics—though not specific to language—centers on the orienting response, reflected as a rapid pupil dilation peaking around 500 ms when attention is captured (Mathôt, 2018). This response tends to be more pronounced for unexpected stimuli (Friedman et al., 1973). From this perspective, the early divergence in pupil size may not only reflect facilitated word recognition in the matched condition, but also an enhanced orienting response in the unmatched condition, where target words are more unexpected given the preceding context. Notably, this initial dilation is sometimes followed by a second phase, typically associated with mental effort or arousal (Mathôt, 2018), which aligns with the later divergences observed in our experiments. Together, these observations support a dynamic, multi-stage account of pupillary responses and highlight the sensitivity of pupillometry to global context effects during word processing.

To better disentangle these overlapping influences, the simultaneous recording of pupil dilation and electroencephalography (EEG) could offer more fine-grained insights. For example, increases in theta-band power (4–7 Hz), particularly in frontal regions, have been linked to increased cognitive control and conflict monitoring (Cavanagh & Frank, 2014), while reductions in alpha-band power (8–12 Hz) have been associated with increased attentional engagement and information processing (Klimesch, 1999). Additionally, event-related potential (ERP) components such as the N400 and the P300 could help differentiate semantic integration from attentional or decisional processes, respectively (Luck, 2014). Thus, studies that coregister pupil size and EEG will allow us to assess if and when these two measures correlate. In fact, some studies showed that pupil size measurements were not correlated with EEG measures, suggesting that they tap into potentially different cognitive mechanisms (McMahon et al., 2016; Miles et al., 2017).

### Limitations and future work

Several unanswered questions remain concerning the specific nature of the global context. For example, recent experiments from our group using the written modality indicate that including only the text but not the image in the global context is sufficient to reproduce the global context effect (Jurado, 2024). Additional studies manipulating other aspects, such as the semantic similarity or the temporal dynamics between the context and the target word, are needed to have a finer-grained measure of when, and for how long, the global context impacts word processing.

In the current study, we showed that the processing of ambiguous words is affected by recent experience, but long-term experiences resulting from different personal interests and life history might also have a strong impact on meaning preferences (Rodd et al., 2016). In a previous study of our group, we have shown that a long-term context that affected the whole population, the COVID-19 pandemic, impacted collective meaning preferences in a word-association task (Laurino et al., 2023). Here, it would be interesting to evaluate if individual life experiences had any impact on meaning availability, and if this accounts for some percentage of the variability observed in our experiments. While the final questionnaire in our study included a general question on personal interest toward each category, as this was not the main aim of our study, we did not include the data in our analyses. In addition, although our sample sizes were sufficient to detect reliable effects at the group level, the relatively small number of participants limits our ability to draw strong conclusions about individual-level variability. Experiments including a more detailed characterization of life experiences and a larger sample size, as well as directly manipulating this factor will be able to clarify individual variability of meaning access.

In summary, we found that semantic context modulates the facilitation and cognitive demands associated with word meaning access. The current results further support the idea that pupillometry provides a valuable neurophysiological window into real-time language processing, in particular, during context-to-meaning integration. Moreover, our results highlight the cognitive system’s flexibility in accessing word meanings and pupil size as a marker of this plasticity. The system adapts dynamically to context and relative meaning frequency, effectively managing ambiguity and facilitating comprehension. This flexibility is crucial for efficient language processing and demonstrates the complex interaction between context and ambiguity in cognitive processing.

## Data Accessibility Statement

All materials, data and scripts are available in OSF https://osf.io/ncugk/?view_only=608c40bd7a2b4418826c7bb789a8057a.

## Additional File

The additional file for this article can be found as follows:

10.5334/joc.454.s1 Appendices. Appendix A to C.
