# Supplementary material for: Pupil Size Tracks the Effects of Global Context and Semantic Ambiguity on Word-Meaning Processing
Source: J Cogn. 2025 Jul 29;8(1):42. doi: 10.5334/joc.454 (PMC12315691; doi:10.5334/joc.454)
Supplement: Appendices. — Appendix A to C. [file joc-8-1-454-s1.pdf]

Appendix A

Ambiguous word	Category	Category relative meaning frequency
raya (stingray)	Animals	0.17
llama (llama)	Animals	0.22
salamandra (salamander)	Animals	0.31
flamenco (flamingo)	Animals	0.49
muñeca (wrist)	Body parts	0.15
anular (ring finger)	Body parts	0.26
tibia (tibia)	Body parts	0.34
palma (palm)	Body parts	0.67
capital (capital)	Economy	0.20
peso (peso)	Economy	0.33
banco (bank)	Economy	0.58
tarjeta (card)	Economy	0.61
granada (pomegranate)	Fruits	0.44
lima (lime)	Fruits	0.47
mango (mango)	Fruits	0.56
naranja (orange)	Fruits	0.66
copa (cup)	Soccer	0.18
amarilla (yellow card)	Soccer	0.19
arco (goal)	Soccer	0.33
penal (penalty)	Soccer	0.48
pico (peak)	Geography	0.10
quebrada (gorge)	Geography	0.30
cabo (cape)	Geography	0.35
sierra (mountain range)	Geography	0.61
sanitario (sanitary)	Medicine	0.17
coma (coma)	Medicine	0.25
cólera (cholera)	Medicine	0.34
paciente (patient)	Medicine	0.61

media (sock)	Clothes	0.43
buzo (hoodie)	Clothes	0.51
sostén (brassiere)	Clothes	0.55
taco (heel)	Clothes	0.58
pluma (pen)	School supplies	0.22
hoja (sheet)	School supplies	0.51
regla (ruler)	School supplies	0.58
goma (eraser)	School supplies	0.63
raíz (root)	Maths	0.13
cuarto (a quarter)	Maths	0.16
medio (half)	Maths	0.17
cuenta (calculation)	Maths	0.38
bajo (bass)	Music	0.18
nota (note)	Music	0.27
órgano (organ)	Music	0.28
batería (drums)	Music	0.30
izquierda (left-wing)	Politics	0.19
partido (party)	Politics	0.20
campaña (campaign)	Politics	0.36
estado (state)	Politics	0.49
papa (pope)	Religion	0.23
pastor (minister)	Religion	0.39
oración (prayer)	Religion	0.43
cura (priest)	Religion	0.50
reparto (cast)	Theater	0.14
función (show)	Theater	0.20
obra (play)	Theater	0.24
público (audience)	Theater	0.48
plataforma (platform)	Technology	0.20
viral (viral)	Technology	0.35
red (Network)	Technology	0.36

aplicación (app)	Technology	0.38
expreso (express)	Transport	0.15
móvil (car)	Transport	0.16
metro (subway)	Transport	0.27
estación (station)	Transport	0.69

**Non-ambiguous word**	**Category**
elefante (elephant)	Animals
ardilla (squirrel)	Animals
oreja (ear)	Body parts
cadera (hip)	Body parts
finanzas (finance)	Economy
moneda (coin)	Economy
limón (lemon)	Fruits
frutilla (strawberry)	Fruits
cancha (field)	Soccer
torneo (championship)	Soccer
montaña (mountain)	Geography
país (country)	Geography
enfermo (sick person)	Medicine
médica (female doctor)	Medicine
remera (T-shirt)	Clothes
polera (sweater)	Clothes
tijera (scissor)	School supplies
sacapuntas (pencil sharpener)	School supplies
álgebra (algebra)	Maths
decimal (decimal)	Maths
trompeta (trumpet)	Music
guitarra (guitar)	Music
presidente (president)	Politics
ministro (minister)	Politics

monja (nun)	Religion
biblia (bible)	Religion
artista (artist)	Theater
actriz (actress)	Theater
teléfono (telephone)	Technology
enchufe (plug)	Technology
tranvía (tram)	Transport
camión (truck)	Transport

Appendix B

Lexical properties of the stimuli used in Experiments 1.

**Word-association task**	Word frequency	Relative meaning frequency	N° letters
Ambiguous words (*n* = 64)			
Mean	91.7	0.35	5.81
SD	139.24	0.17	1.74
Range	0.63 – 713	0.10 – 0.69	3 – 10

Lexical properties of the stimuli used in Experiment 2.

**Semantic relatedness task**	Word frequency	Relative meaning frequency	N° letters	Probe frequency	Probe N° letters
Ambiguous words (*n* = 32)					
Mean	76.51	0.33	5.88	51.52	6.22
SD	94.63	0.16	1.79	76.85	1.54
Range	0.63 – 457	0.10 – 0.69	4 – 10	0.18 – 320	4 – 10
Non-ambiguous words (*n* = 32)					
Mean	77.82	-	6.78	46.41	6.34
SD	204.45	-	1.34	83.46	1.33
Range	0.23 – 912	-	4 – 10	1.56 – 438	4 – 10

Note. Word frequency is per million, as indexed in EsPal - Spanish Lexical Database (Written and Web Tokens [2012-11-06] in Latin American Spanish) (Duchon et al., 2013).

Appendix C

Models used in Experiment 1

Consistency (GLMM, binomial family, logit link)

	Fixed effects	β[1]	p	Random effects[2]
1	context	0.65	**<.001**	Intercepts: participants, words
2	context<br>frequency[3]<br>context × frequency	0.66<br>4.55<br>-0.35	**<.001**<br>**<.001**<br>.65	Intercepts: participants, words

Models used in Experiment 2

Accuracy (GLMM, binomial family, logit link)

	Fixed effects	β[1]	p	Pairwise contrasts matched - unmatched	Random effects[2]
1	context<br>word type<br>context × word type	0.65<br>-1.11<br>0.53	**.001**<br>**.0014**<br>.17	ambiguous:<br>β = 0.82; ***p* < .001**<br>non-ambiguous:<br>β = 0.30, *p* = .36	Intercepts: participants, words
2	context<br>frequency[3]<br>context × frequency	0.81<br>4.90<br>-1.96	**<.001**<br>**.002**<br>.26	-	Intercepts: participants, words

Log-response times (LMM)

	Fixed effects	β	p	Pairwise contrasts matched - unmatched	Random effects[2]
1	context<br>word type<br>context × word type	-0.03<br>0.06<br>0.00	**<.001**<br>**<.001**<br>.89	ambiguous:<br>β = -0.03, ***p* = .01**<br>non-ambiguous:<br>β = -0.04, ***p* = .02**	Intercepts: participants, words ; Slopes: context by words
2	context<br>frequency[3]<br>context × frequency	-0.03<br>-0.16<br>0.27	**.04**<br>**.01**<br>**.004**	-	Intercepts: participants, words; Slopes: context by words

[1] Estimates β are given on the logit (not the response) scale.

[2] The structure of random effects was determined by comparing models with varying random structures with Akaike's Information Criterion and with a Likelihood-Ratio Test, using a forward best-path approach (Barr et al., 2013).

[3] Relative meaning frequency as per estimated during stimuli selection.
